# Effect of pilates exercises plus breastfeeding positioning adjustment on artificial intelligence-assessed craniovertebral angle in breastfeeding women with forward head posture: a randomized controlled trial

**DOI:** 10.1038/s41598-026-63711-9

**Published:** 2026-07-30

**Authors:** Doaa Onsy El blasy, Lama Saad El-Din Mahmoud, Khadiga S. Abdulaziz, Amal Aly El-Taweel, Doaa A. Osman

**Affiliations:** 1 Department of Physical Therapy for Women’s Health, Faculty of Physical Therapy, Cairo University, Giza, Egypt; 2 Department of Physical Therapy for Neurology and Neurosurgery, Faculty of Physical Therapy, October 6 University, Giza, Egypt; 3 Doctorate and Consultant of Pediatrics, International Board-Certified Lactation Consultant, Board member and Director of Education of the Egyptian Lactation Consultants’ Association, Fellow and Board Member of the Academy of Breastfeeding Medicine, Cairo, Egypt

**Keywords:** Breastfeeding, Forward head posture, Craniovertebral angle, Pilates, Breastfeeding positioning, Artificial intelligence, Cervical pain, Health care, Medical research

## Abstract

Forward head posture (FHP), considered a neuromusculoskeletal disorder, includes those associated with breastfeeding. Pilates is recognized for improving posture, flexibility, and core strength. To investigate the effect of Pilates on craniovertebral angle (CVA), cervical spine mobility, pain, neck disability, fatigue, breastfeeding self-efficacy, quality of life (QOL), and infant anthropometric measurements in breastfeeding women with FHP. Seventy‑four breastfeeding women with FHP were randomized into two equal groups. The control group (*n* = 37) received breastfeeding positioning adjustment only, while the intervention group (*n* = 37) received breastfeeding positioning adjustment in addition to Pilates exercises. The Primary outcomes included CVA assessed using Artificial Intelligence Posture Evaluation and Correction System (APECS) and cervical range of motion (ROM), while secondary outcomes included Visual Analogue Scale for pain (VAS-Pain), Neck Disability Index (NDI), Fatigue Severity Scale (FSS), Breastfeeding Self-Efficacy Scale–Short Form (BSES-SF), QOL assessed using Short Form-36 Health Survey (SF-36), and infant anthropometric measurements (weight and length). All outcomes were assessed at the Outpatient Clinic of the Physical Therapy Department, Suez Canal Authority Hospital, Port Said, Egypt, at baseline and after the 8-week intervention. Mixed-design MANOVA demonstrated significant improvements from pre- to post-intervention in both groups across all measured outcomes (all *p* < 0.001). However, the study group exhibited significantly greater improvements than the control group in all outcome measures (all *p* < 0.001). The magnitude of these effects was large to very large, with partial eta-squared values ranging from 0.621 to 0.990. In breastfeeding women with FHP, Pilates exercises with breastfeeding positioning adjustment significantly improve CVA, cervical spine mobility, breastfeeding self-efficacy, QOL, and infant growth parameters, while reducing pain, neck disability, and fatigue.

## Introduction

Musculoskeletal pains are among the common challenges experienced by breastfeeding women^1^. The United Nations Children’s Emergency Fund (UNICEF) and the World Health Organization (WHO) recommend exclusive breastfeeding during the first six months of a child’s life and continued breastfeeding with the introduction of complementary feeding till the age of two years^2^.

Prolonged improper positioning while breastfeeding can disrupt the natural cervical, thoracic, and lumbar spine curves, potentially resulting in chronic deformities and postural syndromes such as hyperlordosis, hyperkyphosis, and, most notably, forward head posture (FHP), as FHP is considered the most prevalent cervical postural abnormality in the sagittal plane^3^. It occurs when the head is protruded anteriorly in relation to the trunk, significantly increasing mechanical stress on cervical structures and leading to a higher risk of neck pain, functional impairment, and considerable socioeconomic burden^4^.

The craniovertebral angle (CVA), which is the angle of the line that crosses through the tragus of the ear and the spinous process of the seventh cervical vertebra relative to the horizontal, is the technique most commonly used to assess head posture^5^, as the flexion of the lower cervical and upper thoracic segments and the extension of the upper cervical spine (C0 to C3 cervical segments) are characteristics of the FHP. This position suggests that the head’s center of gravity is anteriorly displaced with respect to the normal axis of motion for the vertebral column’s flexion and extension^6^. It has been proposed that more thoracic kyphosis may promote a FHP. Additionally, changes in head position may impact other processes such as proprioception, resulting in vertigo, tiredness in other muscles, and deficiencies in coordination^7^.

Therefore, the CVA is a widely accepted clinical parameter for objectively assessing forward head alignment. Using recent advances in artificial intelligence (AI) and computer vision have enabled reliable and accessible measurement of CVA through specialized software^8^. The APECS- (AI Posture Evaluation and Correction System), for instance, offers a user-friendly, cost-effective, and reproducible method for postural screening and research^9^. A posture screen analysis smartphone application that uses automated image processing and landmark detection algorithms to identify anatomical reference points and compute head-neck alignment from standardized digital photographs is used for postural assessment using an artificial intelligence (AI)-based posture screening approach to evaluate CVA, which is considered a quick, non-invasive, and easy-to-use substitute for conventional manual photogrammetry, as the application seeks to reduce examiner-related error and enhance measurement uniformity by eliminating the requirement for manual landmark placement^10^. Previous studies have reported good reproducibility, reliability, and feasibility of APECS for postural evaluation, supporting its use as a standardized and examiner-independent method in both clinical practice and research settings^9,11^. Pilates exercise might improve the stability of the cervical, spinal, lumbar, and pelvic structures and relieve postural abnormalities brought on by muscular asymmetry in FHP^12^.

Pilates exercises are beneficial for maintaining balance, correcting bad posture, and reducing spinal abnormalities. Additionally, it may improve CVA in those with FHP and lessen discomfort and impairment^13^, as it helps the patient become more conscious of how they conduct themselves and can help them stay balanced^14^. Because it strengthens the core, spine, and neck muscles, Pilates also has a strong ability to improve cervical posture, spinal deformity, and participants’ quality of life (QOL). It can help alleviate pain brought on by postural discomfort^12^. Pilates has emerged as a widely practiced form of mind-body exercise focusing on posture, breathing, and coordinated movement^15^.

Although previous researchers have reported the beneficial effects of Pilates exercises for FHP^13,16^, none have investigated the role of Pilates on FHP in breastfeeding women. Also, a variety of therapeutic exercises designed to alleviate the symptoms of FHP have been examined^17^ regardless of the specific causes of the misalignment development. Therefore, this study represents the first investigation to evaluate the influence of Pilates exercises combined with breastfeeding positioning adjustment on APECS-derived CVA measurement, cervical mobility, disability, QOL, fatigue, and pain intensity in breastfeeding women with FHP, addressing a significant gap in maternal musculoskeletal health research. Thus, the primary hypothesis of the current study was that the study group would show greater improvements in CVA, cervical ROM, pain intensity, and neck disability, along with improved fatigue, breastfeeding self-efficacy, and QOL, compared with the control group.

## Materials and methods

### Study design, randomization, and blinding

**Fig. 1:**
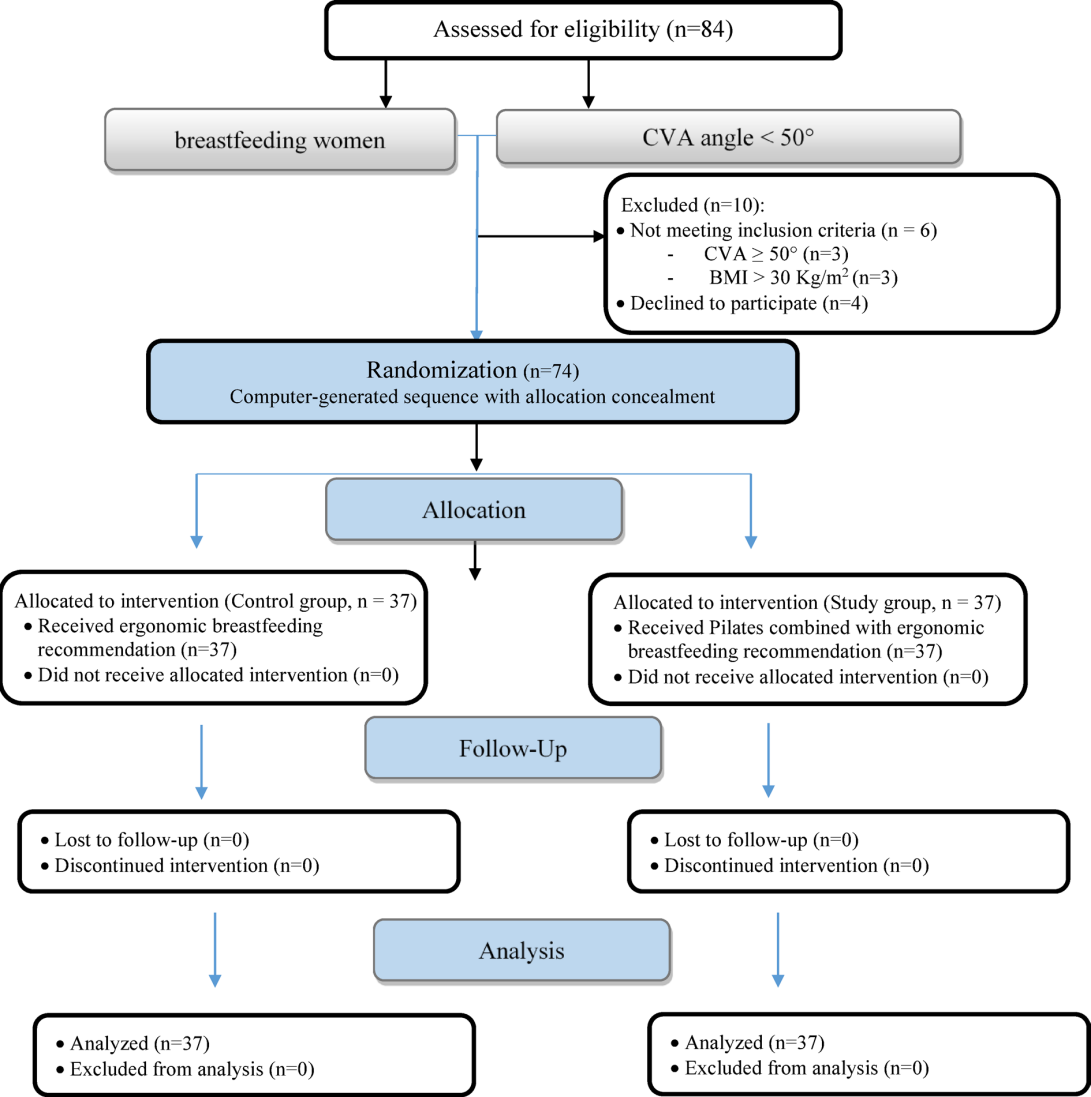
Flow chart showing the experimental design of the study.

A prospective randomized controlled trial was conducted on seventy-four breastfeeding women who were diagnosed with FHP (CVA angle < 50°), confidentiality and anonymity were ensured. Randomization was conducted in accordance with CONSORT guidelines. The allocation sequence was generated by an independent researcher who was not involved in participant recruitment, assessment, treatment, or data analysis using a computer-generated randomization procedure on a Windows-operated computer; patients were assigned at random to one of two equally balanced groups for eight weeks, and until the individual was enrolled and all baseline measurements were finished, group assignments were concealed. Owing to the nature of the exercise intervention, blinding of the participants and the treating physiotherapist was not feasible. However, outcome assessors were blinded to the participants’ group assignment. In addition, data entry and statistical analysis were conducted by a researcher who was not involved in participant recruitment or intervention. Following randomization, there were no reports of research participants dropping out (Fig. 1). Both the control group and the study group received breastfeeding positioning adjustment according to WHO/ UNICEF 2020^2^. In addition, the study group also received Pilates exercises.

### Subjects

All subjects were given written and spoken information about the research prior to enrollment, and they all signed a written consent form, as participants were fully informed of the study’s purpose, procedures, potential risks, and their right to withdraw at any time. All processes were conducted under institutional rules and applicable regulations, guaranteeing confidentiality and anonymity. The requirements for inclusion were as follows: They were diagnosed with FHP (CVA angle < 50°), were six to twelve months after giving birth, and gave birth to a single, healthy, and free of problems infant at full term. They were in the age range of 20 to 35. Their BMI did not exceed 30 kg/m^2^, and their parity varied from two to three times. All participants were housewives and were not employed outside the home. In addition, all previous deliveries had been normal vaginal deliveries. Participants with breast or surrounding cancer, a history of breast or chest surgery, a breast infection, or retracted, cracked, inflamed, or inverted nipples were not allowed to participate. Anemia, diabetes mellitus, recent shoulder or cervical fractures or surgeries, any cardiorespiratory conditions, and no past musculoskeletal, medical or mental health issues were present.

### Ethical approval

 The research involving human participation has been approved by the Faculty of Physical Therapy’s Research Ethics Committee at Cairo University (No: P.T.REC/012/004949). The trial was prospectively registered at ClinicalTrials.gov (Identifier: NCT06271850) on 15 February 2024, before enrollment of the first participant, and participant recruitment commenced on 20 February 2024. The study was conducted in accordance with the Declaration of Helsinki and all applicable national and institutional regulations.

### Sample size calculation

As a previous kind of power analysis for mixed design MANOVA, the sample size was established using the G*Power software version 3.1.9.7 (Heinrich-Heine-Universität Düsseldorf, Düsseldorf, Germany). The CVA is the primary outcome. Based on the mean and standard deviation data from a prior study^13^, which was the most applicable to the present research, an effect size of 0.35 was determined. The study’s power (1-β err prob) was set at 0.8, and the alpha error of probability (α) was set at 0.05. According to the software’s sample size computation, a minimum of 67 people should be included in the sample. To guarantee a high power of significance and to account for 10%–20% sample dropout, the overall sample size should increase to be 74 (37 for each group).

## Methods

### Outcome measures

The primary outcomes included APECS-derived CVA measurement along with cervical range of motion (ROM) in all directions, while the secondary outcomes were: Visual Analogue Scale for pain intensity (VAS-Pain), Neck Disability Index (NDI), Fatigue Severity Scale (FSS), Breastfeeding Self-Efficacy Scale–Short Form (BSES-SF), QOL assessed using Short Form-36 Health Survey (SF-36), and infant anthropometric measurements (weight and length), all parameters evaluated pre and after intervention.

### APECS-derived craniovertebral angle (CVA) measurement

The CVA was measured using the Artificial Intelligence Posture Evaluation and Correction System (APECS^®^, Version 8.5.18; New Body Technologies SAS, Grenoble, France, Android platform), a smartphone-based digital photogrammetry platform developed for computerized postural assessment. By integrating image-processing technology with predefined anatomical landmarks, the application enables objective quantification of postural alignment from standardized digital images. Previous research has demonstrated satisfactory reliability and reproducibility of APECS for postural evaluation, supporting its application as a standardized, examiner-independent assessment tool in both clinical and research environments^9,11^.

To ensure consistency across measurements, all assessments were conducted under standardized conditions between 9:00 a.m. and 11:00 a.m. Participants were instructed to assume a relaxed standing posture while looking at a fixed target positioned at eye level. Sagittal-plane photographs were obtained using the same smartphone and imaging protocol for all participants. During image acquisition, the application’s integrated camera-leveling function provided real-time guidance to ensure proper horizontal alignment of the device. This feature uses a visual indicator that changes color to green once the camera reaches the correct position, thereby helping to reduce measurement errors associated with camera tilt. After image capture, the software guided image cropping to standardize image proportions, and anatomical landmarks were identified according to the predefined application protocol before angle analysis was performed^9^.

**Fig. 2:**
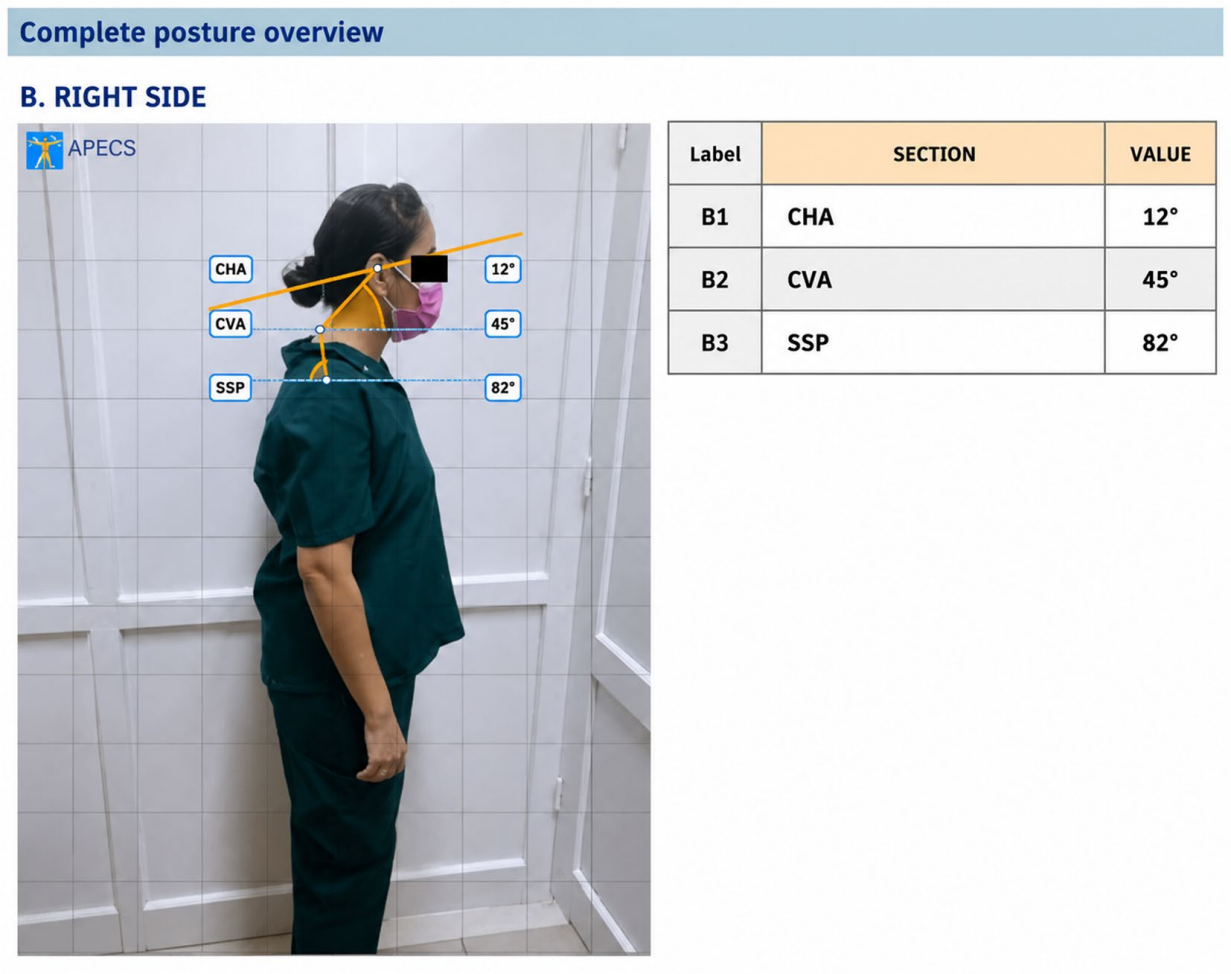
APECS-derived craniovertebral angle (CVA) measurement. The CVA was determined as the angle formed between a horizontal line passing through the spinous process of the seventh cervical vertebra (C7) and a line connecting C7 to the tragus of the ear, as identified by the APECS posture assessment application.

The CVA was calculated using two anatomical reference points: the tragus of the ear and the spinous process of the seventh cervical vertebra (C7). The software automatically determined the angle formed by a horizontal line passing through C7 and a second line connecting the tragus to C7 ^18^. Smaller CVA values indicate a greater degree of FHP, whereas larger values reflect a more neutral head and cervical alignment. A CVA value below 50° was considered indicative of FHP^10^. The APECS-derived CVA measurement is illustrated in Fig. 2.

### Cervical range of motion (ROM)

With patient feet supported on the floor, the subject sat with her back straight, looking forward, and her cervical flexion, extension, lateral flexion, and rotation were measured using a goniometer to determine her cervical ROM. The measurement was taken from “the neutral” position to the final cervical ROM in a specific plane, with the goniometer adjusted for each direction^19^. All cervical ROM measurements were performed by the same trained assessor using a standardized measurement protocol under identical testing conditions throughout the study.

### Neck pain intensity

The Visual Analog Scale for Pain (VAS-Pain), a linear scale that determines the degree of pain severity, was utilized to quantify the intensity of neck pain. It includes a horizontal line that is scaled like a spectrum, with the left end representing minimal discomfort and the right end representing increasing severity. The line is 10 cm long and ends on either side with either severe pain or no discomfort at all. By marking the line on the scale’s spectrum, each participant was asked to rate their present level of pain^20^.

### Neck disability index (NDI)

The NDI, a well-validated 10-item questionnaire covering the following domains: Pain Level, Personal Care, Lifting, Reading, Headaches, Concentration, Work, Driving, Sleeping, and Recreation, was used to assess neck disability. using a 0–5 point rating system for every item. The score ranges from 0 to 50 based on the sum of the 10 components. As explained below, the lower the score, the lower the self-rated disability: No disability is defined as 0–4, mild disability as 5–14, moderate disability as 15–24, severe disability as 25–34, and complete disability as 35 or more^21^.

### Cervical neck muscle fatigue severity scale (FSS)

The Cervical Neck Muscle fatigue was assessed using the Fatigue Severity Scale (FSS), which consists of nine criteria that assess the intensity of fatigue symptoms, was used to measure muscle exhaustion. After reading each statement, the woman marked a number between 1 and 7 that best described her situation. Strong disagreement with the statement is indicated by a low value, whereas strong agreement is indicated by a high value. The participant’s score was calculated by adding all of the numbers she circled. A participant may not be experiencing fatigue if their overall score is less than 36. A participant may be experiencing fatigue if their overall score is 36 or higher^22^.

### Breastfeeding self-efficacy scale-short form (BSES-SF)

The Breastfeeding Self-Efficacy Scale Short Form (BSES-SF), denotes a 14-point self-administered tool originated from the original 33-point BSES that evaluates breastfeeding confidence. A 5-point Likert scale, with 1 denoting “not at all confident” and 5 denoting “always confident,” was used to rate each item. Higher scores indicate more significant levels of breastfeeding self-efficacy; total scores range from 14 to 70. The Arabic version of BSES-SF was used^23^.

### Quality of life short form-36 health survey (SF-36)

The health-related QOL was assessed using the SF-36, a validated generic health survey comprising 36 items distributed across eight domains: physical functioning, role limitations due to physical health, bodily pain, general health, vitality, social functioning, role limitations due to emotional problems, and mental health. Each domain was scored on a scale from 0 to 100, with higher scores indicating better QOL. For the present study, an overall composite score was calculated as the average of the eight domain scores to provide a composite measure of health-related QOL (Brandes et al., 2011)^24^.

### The infant anthropometric measurements (weight and length)

The anthropometric measurements (weight and length) of the infant for each participant were evaluated using the Weighing scale and length board^25^.Infant anthropometric measurements were included as exploratory secondary outcomes because maternal posture and breastfeeding effectiveness may influence infant feeding adequacy and growth. Previous evidence has shown that improving breastfeeding ergonomics enhances infant latch quality and feeding performance, while interventions targeting maternal musculoskeletal function can improve breast milk intake among newborns. Therefore, infant weight and length were assessed as indirect indicators of breastfeeding effectiveness and infant well-being throughout the study period.

### Interventions

Participants in the control group received breastfeeding positioning counseling based on WHO/UNICEF recommendations and were instructed to follow the recommended positioning strategies throughout the study period. Participants in the study group attended supervised Pilates exercise sessions three times per week for eight consecutive weeks in addition to receiving breastfeeding positioning counseling. All exercise sessions were conducted under the direct supervision of a specialized physiotherapist, with no home-based Pilates component included in the intervention. Adherence was monitored using attendance logs, and participants were considered adherent if they attended at least 80% of the scheduled sessions; missed sessions and reasons for non-attendance were documented. Intervention delivery was standardized using a predefined treatment protocol, and both breastfeeding counseling and supervised exercise sessions were delivered by the same physiotherapist at the Outpatient Clinic of the Physical Therapy Department, Suez Canal Authority Hospital, Port Said, Egypt, to ensure consistency. Participants allocated to the control group were instructed not to perform Pilates or engage in any additional neck, postural, or other structured exercise programs during the study period beyond the breastfeeding positioning strategies recommended as part of the study protocol.

### Breastfeeding positioning counseling

**Fig. 3:**
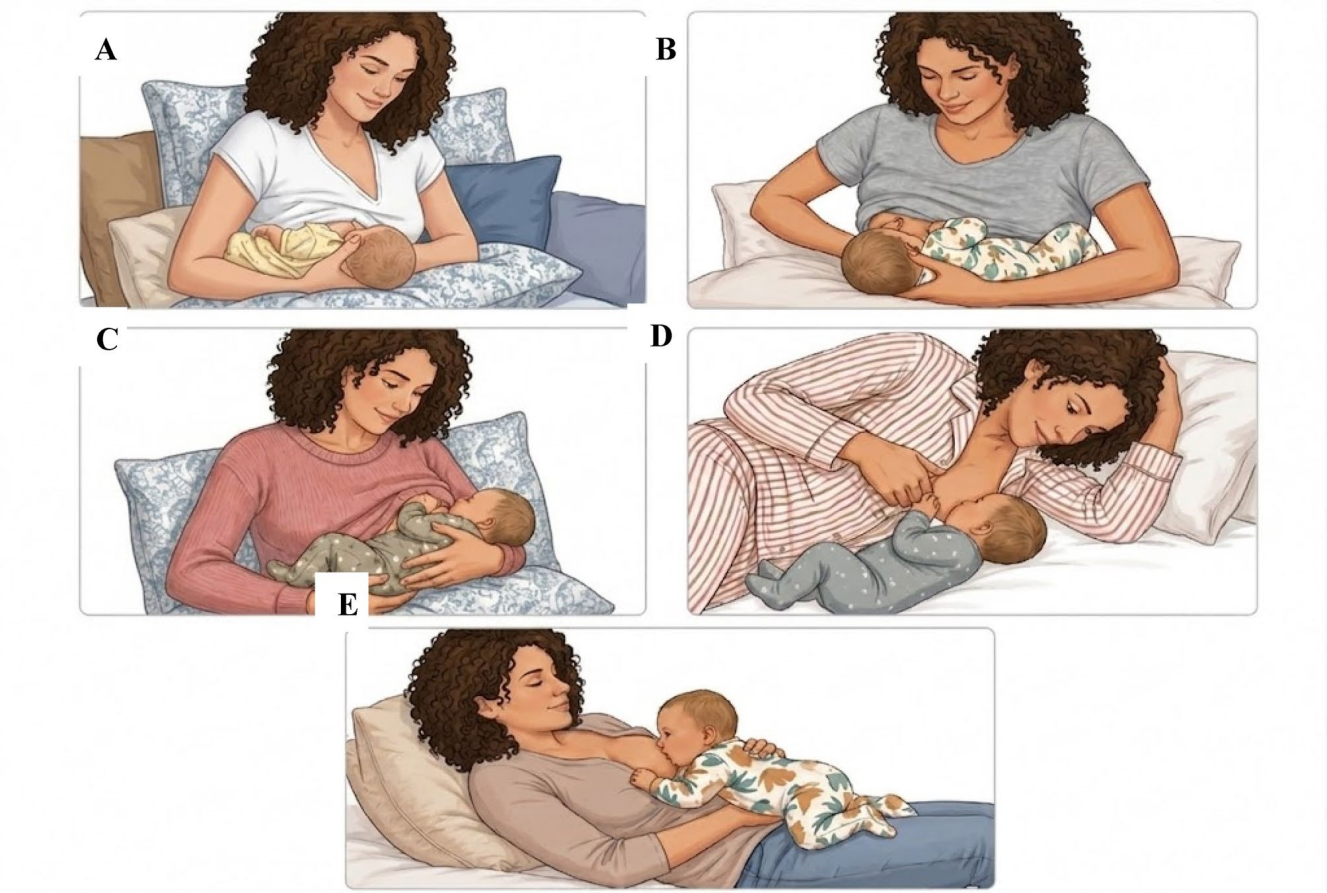
Recommended breastfeeding positions: (**A**) football hold; (**B**) cross‑cradle hold; (**C**) cradle hold; (**D**) side‑lying position; (**E**) laid‑back position.

For eight weeks, all participants in both groups were given breastfeeding positioning adjustments. This adjustment covered the proper positioning and latch on criteria according to the WHO/ UNICEF 2020^2^. These include positioning the mother’s back comfortably supported with slight flexion of the hip before holding the infant, holding the infant’s body supported, close, facing and straight in relation to mother’s body with guiding the mother to bring the infant to her rather than leaning forward towards the infant, using pillows as needed. The following are common breastfeeding positions: cradle hold (same arm as the breast), football hold (under arm) cross cradle hold (opposite arm), side-lying position, and laid-back position^26^, (Fig. 3).

### Pilates exercises

The program incorporated modern Pilates principles targeting FHP-related muscle imbalances through systematic stretching and strengthening protocols. Stretching the neck extensors and pectoral muscles, strengthening the deep neck flexors, shoulder retractors, back, and abdominal muscles, and co-activating the core muscles through breathing technique were the main goals of the Pilates program, which also aimed to balance the primary muscle groups connected to FHP. The participant was told to relax until the pain faded away if movement during the exercise session caused them to feel uncomfortable^13^.

The intervention emphasized proper breathing technique as the foundation, following Joseph Pilates’ methodology with nasal inhalation and controlled oral exhalation through relaxed lips^27^. This breathing pattern facilitated core stability while minimizing neck and shoulder tension.

The Neck stretching exercises were performed in upright sitting/standing positions for the upper trapezius, longissimus capitis, semispinalis capitis, and splenius capitis muscles, also the Pectoral muscle stretching^28^. During weeks 1–2, participants performed 1 set of 20–30-second static stretches for each muscle group; from weeks 3–4 this was increased to 2 sets, and by weeks 5–8 to 3 sets as tolerated. Deep neck flexor strengthening focused on the longus colli and longus capitis muscles through supine chin-nodding exercises^29^. In weeks 1–2, participants completed 1 set of 8–10 repetitions with 5–10-second holds; from weeks 3–4 this progressed to 2 sets of 10 repetitions with 10–15-second holds, and in weeks 5–8 to 3 sets of 10 repetitions with holds up to 20 s, provided technique remained correct. Scapular retractor strengthening included seated scapular adduction exercises and dynamic stabilization movements^30^. Training intensity was progressively increased throughout the intervention period, beginning with 1 set of 10 repetitions and 4-second holds during weeks 1–2, progressing to 2 sets of 10–12 repetitions with 5-second holds during weeks 3–4, and reaching 3 sets of 12 repetitions with 6-second holds and more challenging stabilization tasks during weeks 5–8. A comprehensive core program targeted the anatomical “box”, including rectus abdominis, obliques, erector spinae, and deep stabilizers^31^. During weeks 1–2, participants completed one set of 5–6 repetitions per side, maintaining each position for 3–5 s. In weeks 3–4, the program progressed to two sets of 8–10 repetitions with 5-second holds. Further progression was introduced during weeks 5–8, when participants performed three sets of 10–12 repetitions with hold durations ranging from 5 to 10 s, as long as they could maintain lumbopelvic control. All exercises incorporated specific breathing patterns synchronized with movement phases. Participants were instructed to cease any exercise causing pain and rest until symptoms subsided^13^. The program systematically addressed muscle imbalances associated with FHP through coordinated strengthening of postural muscles and stretching of shortened anterior structures. The structured Pilates exercise protocol, including exercise dosage, progression criteria, and details of intervention delivery, is summarized in Table 1.

**Table 1:** The structured Pilates exercise protocol, including exercise dosage, progression criteria, and details of intervention delivery.

Muscle group target	Exercise	Phase	Sets	Reps / hold	Hold duration	Rest between sets	Progression criteria
Neck extensors & pectorals *Upper trapezius*,* longissimus capitis*,* semispinalis capitis*,* splenius capitis*, pectoralis major/minor	Static stretching	Weeks 1–2	1	1 per muscle	20–30 s	-	Correct technique + no pain over 2 consecutive sessions
	Static stretching	Weeks 3–4	2	1 per muscle	20–30 s	45–60 s	
	Static stretching	Weeks 5–8	3	1 per muscle	20–30 s	45–60 s	
Deep neck flexors *Longus colli*, longus capitis	Supine chin-nodding	Weeks 1–2	1	8–10 reps	5–10 s	-	
	Supine chin-nodding	Weeks 3–4	2	10 reps	10–15 s	45–60 s	
	Supine chin-nodding	Weeks 5–8	3	10 reps	Up to 20 s	45–60 s	
Scapular retractors *Rhomboids*, middle trapezius	Seated scapular adduction; dynamic stabilization	Weeks 1–2	1	10 reps	4 s	-	
	Seated scapular adduction; dynamic stabilization	Weeks 3–4	2	10–12 reps	5 s	45–60 s	
	Seated scapular adduction; dynamic stabilization	Weeks 5–8	3	12 reps	6 s	45–60 s	
Core stabilizers *Rectus abdominis*,* obliques*,* erector spinae*, deep stabilizers	Pelvic bridging; quadruped / bird-dog	Weeks 1–2	1	5–6 reps/side	3–5 s	-	
	Pelvic bridging; quadruped / bird-dog; modified kneeling	Weeks 3–4	2	8–10 reps/side	5–8 s	45–60 s	
	Pelvic bridging; quadruped / bird-dog; modified kneeling; superman variations	Weeks 5–8	3	10–12 reps/side; superman 5–8 reps	8–10 s	45–60 s	

Breathing foundation: All exercises incorporated specific breathing patterns synchronized with movement phases.

Supervision: Individual supervised sessions conducted by the same physiotherapist.

Setting: The Outpatient Clinic of the Physical Therapy Department, Suez Canal Authority Hospital, Port Said, Egypt.

Frequency: 3 sessions/week

Total treatment duration: 8 weeks

### Data exploration for normality

Prior to the main statistical analyses, all data were screened for accuracy, completeness, and compliance with the assumptions underlying parametric statistical procedures. The distribution of each continuous variable was examined using the Shapiro–Wilk test, which is considered one of the most robust and appropriate methods for assessing normality, particularly in studies with small to moderate sample sizes. The results of the Shapiro–Wilk test indicated that all outcome variables were normally distributed (*p* > 0.05), supporting the assumption of normality required for subsequent parametric analyses. In addition, visual inspection of histograms and Q–Q plots confirmed the absence of substantial deviations from a normal distribution.

### Statistical analysis

To ensure comparability of the two groups before the intervention, independent-samples t-tests were performed to compare baseline demographic and clinical characteristics between the control and study groups. Variables examined included age, body mass index (BMI), parity, infant age at baseline, and daily breastfeeding duration. The independent-samples t-test was selected because the compared variables were continuous, normally distributed, and measured in two independent groups.

The effects of the intervention on the twelve outcome measures were examined using a two-way mixed-design multivariate analysis of variance (Mixed MANOVA). This statistical approach was selected because the study involved one between-subject factor (group: study versus control) and one within-subject factor (time: pre-treatment versus post-treatment), with multiple continuous dependent variables assessed simultaneously. Mixed MANOVA is particularly advantageous in this context because it accounts for the correlations among the outcome variables while evaluating them within a single multivariate framework. Given that the twelve dependent variables represent related dimensions of patient performance and function, separate univariate analyses for each outcome would not only ignore the covariance structure among variables but would also substantially increase the risk of alpha inflation and Type I error due to multiple statistical testing. By analyzing all dependent variables simultaneously, Mixed MANOVA effectively controls the experiment-wise error rate, thereby reducing the likelihood of false-positive findings while preserving statistical power. Furthermore, this approach provides a comprehensive evaluation of treatment effects through the simultaneous assessment of the main effect of time, the main effect of group, and the group-by-time interaction effect. The interaction effect was of primary interest because it determines whether the magnitude of change over time differed significantly between the study and control groups. Therefore, the use of Mixed MANOVA was methodologically and statistically justified for evaluating the multidimensional impact of the intervention across the twelve correlated outcome measures while minimizing alpha inflation and maintaining the integrity of statistical inference. Statistical significance was established at *p* < 0.05 for all analyses. All statistical procedures were conducted using IBM Statistical Package for Social Sciences (SPSS) version 28.0 for Windows.

## Results

### Intervention adherence

**Fig. 4:**
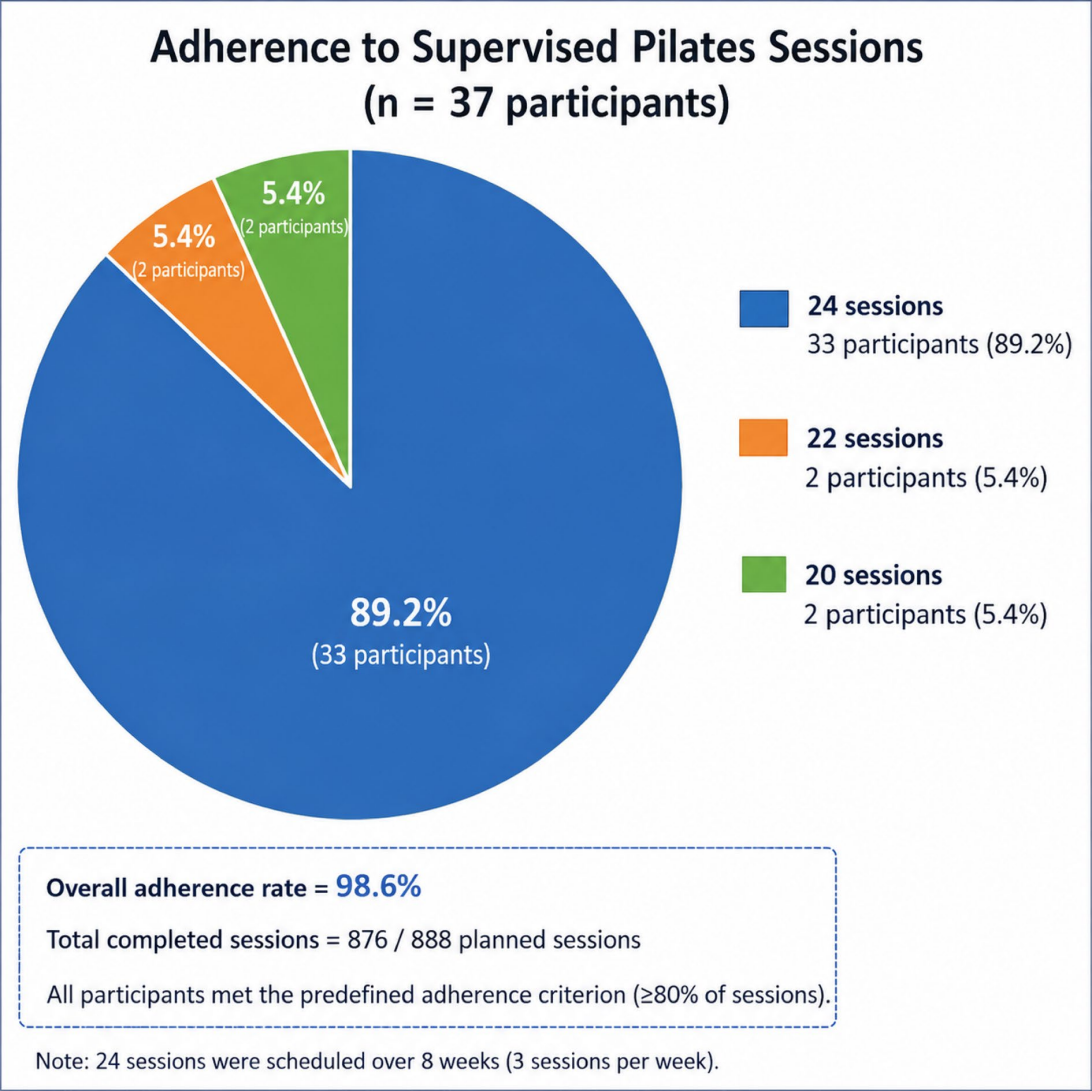
Adherence to the supervised Pilates intervention during the 8-week study period.

Adherence to the supervised Pilates intervention was high (Fig. 4). The overall adherence rate was 98.6%, with 33 participants attending all 24 scheduled sessions, two participants attending 22 sessions, and two participants attending 20 sessions. Consequently, all participants met the predefined adherence criterion of attending at least 80% of the scheduled sessions.

### Comparison of participants’ baseline demographic and clinical characteristics between the control and study groups

Independent-samples t-test was conducted to compare baseline demographic and clinical characteristics between the control group (A, *n* = 37) and the study group (B, *n* = 37). The analysis revealed no statistically significant differences between the two groups regarding age (*p* = 0.465), body mass index (BMI) (*p* = 0.606), parity (*p* = 0.055), infant age at baseline (*p* = 0.310), or daily breastfeeding duration (*p* = 0.864). These findings indicate that the two groups were comparable at baseline, confirming the homogeneity of the sample and minimizing the potential influence of confounding variables on the study outcomes (Table 2).

**Table 2:** participants’ baseline characteristics for the two tested groups.

Variables	(X ± SD)		*P*-value
	Control group (*n* = 37)	Study group (*n* = 37)	
Age (years)	29.62 ± 3.83	28.97 ± 3.77	0.465
BMI (kg/m²)	27.18 ± 1.74	26.96 ± 1.88	0.606
Parity (times)	2.26 ± 0.70	2.56 ± 0.63	0.055
Infant age at baseline (days)	273.19 ± 56.83	257.65 ± 64.92	0.310
Daily breastfeeding duration (minutes)	149.24 ± 24.17	150.22 ± 24.60	0.864

All participants had normal vaginal deliveries according to the predefined inclusion criteria.

$\overline{X}$, mean;SD, standard deviation; BMI, body mass index.

### Descriptive statistics of all outcome measures of the two tested groups

Table 3 shows descriptive statistics demonstrating improvements in all outcome measures in both groups after the intervention. Greater changes were observed in the study group. Regarding cervical posture, the CVA increased from 46.94 ± 0.96° to 51.23 ± 1.20° in the study group, representing a 9.14% improvement, compared with an increase from 47.19 ± 0.97° to 49.23 ± 1.05° in the control group (4.32% improvement). Pain intensity VAS scores, decreased by 65.66% in the study group (7.60 ± 1.30 cm to 2.61 ± 1.11 cm) compared with 44.34% in the control group (7.60 ± 1.28 cm to 4.23 ± 1.03 cm). Similarly, NDI scores decreased by 36.91% in the study group (39.88 ± 1.54 to 25.16 ± 4.21) and by 22.42% in the control group (40.14 ± 1.58 to 31.14 ± 2.44).

**Table 3:** Descriptive statistics of the outcome measures in the two tested groups.

Outcome measures	Time	Control group (*n* = 37)	Study group (*n* = 37)
CVA (degrees)	Pre	47.19 ± 0.97	46.94 ± 0.96
	Post	49.23 ± 1.05	51.23 ± 1.20
VAS	Pre	7.60 ± 1.28	7.60 ± 1.30
	Post	4.23 ± 1.03	2.61 ± 1.11
NDI	Pre	40.14 ± 1.58	39.88 ± 1.54
	Post	31.14 ± 2.44	25.16 ± 4.21
Flexion ROM (degrees)	Pre	63.41 ± 4.24	64.14 ± 3.52
	Post	67.62 ± 3.82	78.22 ± 2.73
Extension ROM (degrees)	Pre	43.49 ± 3.14	44.65 ± 4.65
	Post	52.28 ± 3.81	65.64 ± 2.69
Right lateral flexion ROM (degrees)	Pre	25.81 ± 4.39	26.92 ± 3.72
	Post	34.22 ± 4.50	40.96 ± 3.43
Left lateral flexion ROM (degrees)	Pre	26.95 ± 3.90	25.81 ± 4.39
	post	35.76 ± 3.77	40.61 ± 3.98
FSS	Pre	46.22 ± 5.16	46.14 ± 6.05
	Post	36.97 ± 2.89	31.14 ± 3.43
BSES-SF	Pre	31.30 ± 7.53	31.41 ± 7.42
	Post	43.54 ± 5.07	57.41 ± 5.47
SF 36	Pre	40.19 ± 7.22	40.00 ± 6.03
	Post	60.89 ± 5.30	83.53 ± 4.76
Infant length (cm)	Pre	70.10 ± 3.02	70.23 ± 2.95
	Post	71.78 ± 2.83	74.78 ± 2.48
Infant weight (Kg)	Pre	8.62 ± 1.13	8.59 ± 1.07
	Post	9.62 ± 0.90	10.16 ± 0.68

$\overline{X}$®, mean; SD, standard deviation; CI, confidence interval; η², partial eta squared; CVA, craniovertebral angle; VAS, Visual Analogue Scale; NDI, Neck Disability Index; ROM, range of motion; FSS, Fatigue Severity Scale; BSES-SF, Breastfeeding Self-Efficacy Scale–Short Form; SF-36, 36-Item Short Form Health Survey. Significant at α < 0.05.

Cervical ROM showed significant improvements in both groups, with significantly greater improvements in the study group. Cervical flexion ROM increased from 64.14 ± 3.52° to 78.22 ± 2.73° in the study group, corresponding to a 21.95% improvement, whereas the control group improved from 63.41 ± 4.24° to 67.62 ± 3.82° (6.64% improvement). Cervical extension ROM increased from 44.65 ± 4.65° to 65.64 ± 2.69° in the study group (47.01% improvement) compared with an increase from 43.49 ± 3.14° to 52.28 ± 3.81° in the control group (20.21% improvement). Right lateral flexion ROM increased from 26.92 ± 3.72° to 40.96 ± 3.43° in the study group (52.15% improvement), while the control group improved from 25.81 ± 4.39° to 34.22 ± 4.50° (32.58% improvement). Similarly, left lateral flexion ROM increased from 25.81 ± 4.39° to 40.61 ± 3.98° in the study group (57.34% improvement), compared with an increase from 26.95 ± 3.90° to 35.76 ± 3.77° in the control group (32.69% improvement).

FSS scores decreased by 32.51% in the study group (46.14 ± 6.05 to 31.14 ± 3.43) compared with 20.01% reduction in the control group (46.22 ± 5.16 to 36.97 ± 2.89).Substantial improvements were also observed in breastfeeding (BSES-SF scores), which increased by 82.78% in the study group (31.41 ± 7.42 to 57.41 ± 5.47) compared with 39.11% in the control group (31.30 ± 7.53 to 43.54 ± 5.07). Likewise, SF-36 scores improved by 108.82% in the study group (40.00 ± 6.03 to 83.53 ± 4.76) and by 51.51% in the control group (40.19 ± 7.22 to 60.89 ± 5.30).

In terms of growth outcome in infants, the length of the babies in the study group increased from 70.23 ± 2.95 cm to 74.78 ± 2.48 cm, with a percentage increase of 6.48%, while babies in the control group increased their length from 70.10 ± 3.02 cm to 71.78 ± 2.83 cm, with a percentage increase of 2.40%. Similarly, infant weight increased from 8.59 ± 1.07 kg to 10.16 ± 0.68 kg in the study group (18.28% increase), compared with an increase from 8.62 ± 1.13 kg to 9.62 ± 0.90 kg in the control group (11.60% increase). Overall, the descriptive findings indicate superior post-treatment improvements in the study group across all measured outcomes.

### Within-group comparisons of outcome measures

Two-way mixed design MANOVA pairwise comparisons with Bonferroni adjustment demonstrated that there were significant improvements from pre- to post-intervention in both the control and study groups across all outcome measures (all *p* < 0.001) (Table 4).

**Table 4:** within group pairwise comparison in each tested group.

Outcome measures	F value	Effect size (η²)	Groups	Pre-intervention vs. post-intervention	
				MD (95% CI)	P value
CVA (degrees)	638.302	0.899	Control	2.40 (1.69:2.39)	*P* < 0.001*
			Study	4.29 (3.94:4.65)	*P* < 0.001*
VAS (cm)	1094.89	0.938	Control	3.38 (3.02:3.73)	*P* < 0.001*
			Study	4.99 (4.64:5.35)	*P* < 0.001*
NDI	1364.16	0.950	Control	9.00 (8.09:9.90)	*P* < 0.001*
			Study	14.72 (13.81:15.62)	*P* < 0.001*
Flexion ROM (degrees)	1079.69	0.937	Control	4.22 (3.43:5.00)	*P* < 0.001*
			Study	14.08 (13.30:14.87)	*P* < 0.001*
Extension ROM (degrees)	2149.03	0.968	Control	8.79 (7.89:9.70)	*P* < 0.001*
			Study	21.00 (20.09:21.90)	*P* < 0.001*
Right lateral flexion ROM (degrees)	1964.31	0.965	Control	8.41 (7.69:9.12)	*P* < 0.001*
			Study	14.04 (13.32:14.75)	*P* < 0.001*
Left lateral flexion ROM (degrees)	1285.521	0.947	Control	8.81 (7.88:9.73)	*P* < 0.001*
			Study	14.80 (13.87:15.73)	*P* < 0.001*
FSS	1019.32	0.934	Control	9.24 (8.17:10.31)	*P* < 0.001*
			Study	14.99 (13.92:16.06)	*P* < 0.001*
BSEF-SF	979.80	0.932	Control	12.24 (10.52:13.70)	*P* < 0.001*
			Study	26.01 (24.29:27.73)	*P* < 0.001*
SF 36	7354.243	0.990	Control	20.70 (19.65:21.76)	*P* < 0.001*
			Study	43.53 (42.47:44.58)	*P* < 0.001*
Infant length (cm)	117.75	0.621	Control	1.69 (0.88:2.50)	*P* < 0.001*
			Study	4.55 (3.74:5.37)	*P* < 0.001*
Infant weight (Kg)	659.61	0.902	Control	1.00 (0.85:1.13)	*P* < 0.001*
			Study	1.57 (1.43:1.71)	*P* < 0.001*

$\overline{X}$, mean; SD, standard deviation; CI, confidence interval; η², partial eta squared; CVA, craniovertebral angle; VAS, Visual Analogue Scale; NDI, Neck Disability Index; ROM, range of motion; FSS, Fatigue Severity Scale; BSES-SF, Breastfeeding Self-Efficacy Scale–Short Form; SF-36, 36-Item Short Form Health Survey. Significant at α < 0.05.

In the control group (A), CVA improved significantly with a mean difference (MD) of 2.40 (95% CI: 1.69–2.39; F = 638.302, η² = 0.899, *p* < 0.001). Significant reductions were also observed in pain intensity (VAS) and NDI scores, with MDs of 3.38 (95% CI: 3.02–3.73; F = 1094.89, η² = 0.938, *p* < 0.001) and 9.00 (95% CI: 8.09–9.90; F = 1364.16, η² = 0.950, *p* < 0.001), respectively. Cervical ROM improved significantly in all directions, with MDs of 4.22 (95% CI: 3.43–5.00; F = 1079.69, η² = 0.937, *p* < 0.001) for flexion, 8.79 (95% CI: 7.89–9.70; F = 2149.03, η² = 0.968, *p* < 0.001) for extension, 8.41 (95% CI: 7.69–9.12; F = 1964.31, η² = 0.965, *p* < 0.001) for right lateral flexion, and 8.81 (95% CI: 7.88–9.73; F = 1285.521, η² = 0.947, *p* < 0.001) for left lateral flexion.

Significant improvements were also observed for FSS (MD = 9.24, 95% CI: 8.17–10.31; F = 1019.32, η² = 0.934, *p* < 0.001), BSEF-SF (MD = 12.24, 95% CI: 10.52–13.70; F = 979.80, η² = 0.932, *p* < 0.001), SF 36 (MD = 20.70, 95% CI: 19.65–21.76; F = 7354.243, η² = 0.990, *p* < 0.001), infant length (MD = 1.69, 95% CI: 0.88–2.50; F = 117.75, η² = 0.621, *p* < 0.001), and infant weight (MD = 1.00, 95% CI: 0.85–1.13; F = 659.61, η² = 0.902, *p* < 0.001).

Likewise, the study group (B) showed statistically significant pre-to post intervention gains for all outcomes. CVA improved by 4.29 (95% CI: 3.94–4.65; F = 638.302, η² = 0.899, *p* < 0.001). Significant reductions were also observed for pain intensity VAS scores (MD = 4.99, 95% CI: 4.64–5.35; F = 1094.89, η² = 0.938, *p* < 0.001) and NDI scores (MD = 14.72, 95% CI: 13.81–15.62; F = 1364.16, η² = 0.950, *p* < 0.001). Cervical ROM increased significantly, with MDs of 14.08 (95% CI: 13.43–14.87; F = 1079.69, η² = 0.937, *p* < 0.001) for flexion, 21.00 (95% CI: 20.09–21.90; F = 2149.03, η² = 0.968, *p* < 0.001) for extension, 14.04 (95% CI: 13.32–14.75; F = 1964.31, η² = 0.965, *p* < 0.001) for right lateral flexion, and 14.80 (95% CI: 13.87–15.73; F = 1285.521, η² = 0.947, *p* < 0.001) for left lateral flexion.

Significant improvements were further demonstrated in FSS (MD = 14.99, 95% CI: 13.92–16.06; F = 1019.32, η² = 0.934, *p* < 0.001), BSEF-SF (MD = 26.01, 95% CI: 24.29–27.73; F = 979.80, η² = 0.932, *p* < 0.001), SF 36 (MD = 43.53, 95% CI: 42.47–44.58; F = 7354.243, η² = 0.990, *p* < 0.001), infant length (MD = 4.55, 95% CI: 3.74–5.37; F = 117.75, η² = 0.621, *p* < 0.001), and infant weight (MD = 1.57, 95% CI: 1.43–1.71; F = 659.61, η² = 0.902, *p* < 0.001) (Table 4).

Table 4**is here**.

### Between-group comparisons of outcome measures

As revealed by Tukey post hoc pairwise comparisons, there were no significant differences between the control group (A) and the study group (B) at baseline for any outcome measure (all *p* > 0.05). This confirms the homogeneity and comparability of the two groups before treatment (Table 5).

**Table 5:** between group pairwise comparison in each tested time.

Outcome measures	F value	Effect size (η²)	Testing time	Control group vs. Study group	
				MD (95% CI)	P value
CVA (degrees)	17.70	0.197	Pre	0.250(-0.20:0.70)	0.268
			Post	2 (1.48:2.53)	*P* < 0.001*
VAS (cm)	11.05	0.133	Pre	3.07 (-0.59:0.59)	1.000
			Post	1.62(1.12:2.12)	*P* < 0.001*
NDI	34.35	0.323	Pre	0.26(-0.47:0.98)	0.479
			Post	5.98(4.38:7.57)	*P* < 0.001*
Flexion ROM (degrees)	50.85	0.414	Pre	0.73(-1.08:2.54)	0.423
			Post	10.98(9.06:12.13)	*P* < 0.001*
Extension ROM (degrees)	85.52	0.543	Pre	1.16(-0.68:3.0)	0.213
			Post	13.37(11.84:14.89)	*P* < 0.001*
Right lateral flexion ROM (degrees)	18.91	0.208	Pre	1.11(-0.77:3.0)	0.243
			Post	6.74(4.89:8.60)	*P* < 0.001*
Left lateral flexion ROM (degrees)	4.51	0.059	Pre	1.14(-0.78:3.07)	0.241
			Post	4.85(3.06:6.65)	*P* < 0.001*
FSS	8.88	0.110	Pre	0.08(-2.53:2.69)	0.951
			Post	5.83(4.36:7.30)	*P* < 0.001*
BSEF-SF	25.89	0.264	Pre	0.11(-3.36:3.57)	0.951
			Post	13.87(11.43:16.32)	*P* < 0.001*
SF 36	72.42	0.501	Pre	0.20(-2.89:3.27)	0.903
			Post	22.64(20.30:24.97)	*P* < 0.001*
Infant length (cm)	7.02	0.089	Pre	0.132(-1.25:1.52)	0.850
			Post	3(1.77:4.23)	*P* < 0.001*
Infant weight (Kg)	1.37	0.019	Pre	0.03(-0.48:0.54)	0.900
			Post	0.54(0.17:0.91)	0.005*

$\overline{X}$, mean; SD, standard deviation; CI, confidence interval; η², partial eta squared; CVA, craniovertebral angle; VAS, Visual Analogue Scale; NDI, Neck Disability Index; ROM, range of motion; FSS, Fatigue Severity Scale; BSES-SF, Breastfeeding Self-Efficacy Scale–Short Form; SF-36, 36-Item Short Form Health Survey. Significant at α < 0.05.

At the pre-intervention assessment, no significant between-group differences were observed for CVA [MD = 0.25, 95% CI (− 0.20:0.70), *p* = 0.268], VAS [MD = 3.07, 95% CI (− 0.59:0.59), *p* = 1.000], NDI [MD = 0.26, 95% CI (− 0.47:0.98), *p* = 0.479], flexion ROM [MD = 0.73, 95% CI (− 1.08:2.54), *p* = 0.423], extension ROM [MD = 1.16, 95% CI (− 0.68:3.00), *p* = 0.213], right lateral flexion ROM [MD = 1.11, 95% CI (− 0.77:3.00), *p* = 0.243], left lateral flexion ROM [MD = 1.14, 95% CI (− 0.78:3.07), *p* = 0.241], FSS [MD = 0.08, 95% CI (− 2.53:2.69), *p* = 0.951], BSES-SF [MD = 0.11, 95% CI (− 3.36:3.57), *p* = 0.951], SF-36 [MD = 0.20, 95% CI (− 2.89:3.27), *p* = 0.903], infant length [MD = 0.13, 95% CI (− 1.25:1.52), *p* = 0.850], or infant weight [MD = 0.03, 95% CI (− 0.48:0.54), *p* = 0.900].

Given the significant group × time interaction (reported in the MANOVA), post-hoc pairwise comparisons were conducted to examine between-group differences at post-intervention.

At the post-intervention, significant higher scores were detected in the study group than the control group with significant between-group differences across all measured outcomes (all *p* < 0.05). CVA was significantly higher in the study group than in the control group [MD = 2.00, 95% CI (1.48:2.53), F = 17.70, η² = 0.197, *p* < 0.001]. Significant differences were also observed for VAS [MD = 1.62, 95% CI (1.12:2.12), F = 11.05, η² = 0.133, *p* < 0.001] and NDI [MD = 5.98, 95% CI (4.38:7.57), F = 34.35, η² = 0.323, *p* < 0.001]. Cervical ROM outcomes showed significant advantages for the study group, including flexion ROM [MD = 10.98, 95% CI (9.06:12.13), F = 50.85, η² = 0.414, *p* < 0.001], extension ROM [MD = 13.37, 95% CI (11.84:14.89), F = 85.52, η² = 0.543, *p* < 0.001], right lateral flexion ROM [MD = 6.74, 95% CI (4.89:8.60), F = 18.91, η² = 0.208, *p* < 0.001], and left lateral flexion ROM [MD = 4.85, 95% CI (3.06:6.65), F = 4.51, η² = 0.059, *p* < 0.001].

Furthermore, the study group demonstrated significantly better outcomes in FSS [MD = 5.83, 95% CI (4.36:7.30), F = 8.88, η² = 0.110, *p* < 0.001], BSEF-SF [MD = 13.87, 95% CI (11.43:16.32), F = 25.89, η² = 0.264, *p* < 0.001], and QOL [MD = 22.64, 95% CI (20.30:24.97), F = 72.42, η² = 0.501, *p* < 0.001]. Significant between-group differences were also found for infant growth outcomes, with greater improvements in infant length [MD = 3.00, 95% CI (1.77:4.23), F = 7.02, η² = 0.089, *p* < 0.001] and infant weight [MD = 0.54, 95% CI (0.17:0.91), F = 1.37, η² = 0.019, *p* = 0.005] in the study group compared with the control group (Table 5).

## Discussion

The purpose of the current study was to examine how Pilates exercises affect breastfeeding women’s FHP using CVA, NDI, VAS, cervical ROM, FSS, (BSES-SF), SF-36, weight, and length of the infant. The present study’s main finding showed that both Pilates and breastfeeding positioning adjustment groups had statistically significant improvements from baseline to 8 weeks post-intervention, as there was a significant increase in CVA, ROM of cervical, BSES-SF, SF-36, weight, and length of the infant, also, there was a significant reduction in VAS, NDI, and FSS post-treatment in study group more than control group.

The substantial effect sizes observed in the present study may be attributed to several methodological and clinical factors, including the clinically meaningful baseline impairments, the relatively homogeneous study population resulting from the strict eligibility criteria, and the use of well-validated, responsive outcome measures. In addition, the use of a non-active control group may have contributed to larger between-group effect size estimates through non-specific effects such as therapist attention and supervised intervention. Therefore, the observed effect sizes should be interpreted within the methodological context of the present study.

The current study revealed a significantly higher improvement in the study group, reflecting the beneficial effect of Pilates exercise in treating FHP, this result was supported by Lee et al.^13^, who investigated the clinical effectiveness of a Pilates treatment for FHP, the study found that there were significant increases in CVA and cervical ROM and decreased VAS and NDI in the Pilates group. Similarly, Shah and Soni^32^ examined the influence of Pilates in conjunction with a traditional training program in individuals with text neck syndrome. They found that, in comparison to the conventional exercise program alone, there was a noticeably higher improvement in pain reduction, neck disability, and cervical muscle strength and endurance.

The prevalence of breastfeeding-related neuromusculoskeletal problems as cervical pain, is high up to more than 50% across different populations, as shown by previous studies ^33,34^. The repeated forward-leaning postures adopted while breastfeeding can significantly exacerbate FHP and contribute to these high prevalence rates^33^. Additionally, pregnancy-related alterations in sagittal spinal alignment, particularly increased thoracic kyphosis and lumbar lordosis, may persist beyond childbirth and have been associated with reduced craniovertebral angle, neck pain, and functional limitations^35,36^. The biomechanical demands of breastfeeding may further amplify these changes, as prolonged and repetitive feeding postures have been linked to sustained activation of the cervical and shoulder muscles^37^. Moreover, the combined influence of residual postpartum adaptations and the physical demands of infant care may increase susceptibility to postural disorders such as upper crossed syndrome among lactating women^38,39^. Interpreting these findings within the postpartum period highlights the multifactorial nature of maternal musculoskeletal strain, arising from the interaction between persistent post-pregnancy anatomical adaptations and the considerable physical demands of infant care^40^.

Within this postpartum and breastfeeding framework, the present findings extend the existing evidence by suggesting that a Pilates program emphasizing lumbopelvic and scapular stabilization, delivered alongside breastfeeding positioning counseling, may help improve FHP and associated musculoskeletal complaints in breastfeeding women. The results are supported by Li et al.^12^, who observed that Pilates techniques improve QOL, pain alleviation, function, and fitness while also effectively correcting posture and spinal abnormalities. Additionally, it has been demonstrated that Pilates greatly enhances spinal range of motion and decreases the Cobb angle.

Behavioral factors may contribute to the development of FHP. People may continue to strain themselves for extended periods of time because they are unaware of their poor posture^13^, hence, the results of this study agreed with Elhafez et al.^41^, who compared the effects of Pilates and cervical stabilization exercises on the cervical muscles’ CVA, pain, function, and myoelectrical activity in FHP. The findings indicated that Pilates exercises improve CVA, strengthen the deep neck musculature, and lead to whole-body muscle rehabilitation, which in turn alleviates neck pain.

The magnitude of the between-group differences observed in the present study suggests that the benefits of Pilates training may be clinically meaningful in addition to being statistically significant. Regarding postural alignment, the Pilates group exhibited a 2° greater improvement in CVA (95% CI 1.48–2.53) than the control group. Notably, this change exceeded the established minimal clinically important difference (MCID) of 1.40° for CVA correction^42^, supporting the clinical relevance of the postural adaptations achieved through the intervention. In addition, participants in the Pilates group demonstrated a 1.62-cm greater reduction in pain intensity on the VAS (95% CI 1.12–2.12) compared with controls. This improvement exceeded the reported MCID of 1.37 cm, indicating that the reduction in pain was likely perceptible and meaningful from the patient’s perspective^43^. Similarly, the between-group difference in NDI scores reached 5.98 points (95% CI 4.38–7.57), surpassing the reported minimal clinically important change threshold of approximately 3–3.5 points for patients with neck pain^43,44^, this finding suggests that the observed improvement in neck-related disability was sufficiently large to be considered clinically meaningful. Collectively, these findings suggest that Pilates training may produce clinically relevant improvements in posture, pain, and functional disability among breastfeeding women with FHP. However, it should be acknowledged that these published MCID values were derived from neck pain populations rather than breastfeeding women with forward head posture and therefore provide supportive clinical context rather than population-specific thresholds.

Nevertheless, these findings should be interpreted with appropriate caution. Although the intervention group demonstrated superior outcomes, the study was designed to evaluate the effectiveness of adding a supervised Pilates program to standard breastfeeding positioning education rather than to isolate the specific physiological effects of Pilates. Because the control group did not receive an attention-matched supervised intervention, part of the observed between-group differences may have been influenced by non-specific factors such as increased therapist interaction, supervised contact, or general physical activity. Therefore, future randomized controlled trials incorporating attention-matched active comparator interventions are warranted to better distinguish the intervention-specific effects of Pilates.

Pilates training is specifically planned to enhance overall body flexibility and well-being, emphasizing core strength, posture, and synchronized breathing. It’s adaptable for individual needs and can effectively address specific postural issues. Research suggests that Pilates can have a significant positive impact on cervical spine health, with studies demonstrating significant improvements in CVA, cervical ROM, and postural alignment in individuals with FHP^16^.

Workplace support and mothers’ self-efficacy are significant factors in determining breastfeeding practice among working women, according to the results of previous research^45^, which used the breastfeeding Self Efficacy Scale Short Form (BSES-SF), as these factors were found to be positively correlated.

Ahmed et al.^46^ found a correlation between breastfeeding and infant growth measured by weight and length, and Moradi et al.^47^ discovered that exclusive breastfeeding with breast milk has a significant impact on the child’s growth indicators. These previous studies provide a clinical context for including infant anthropometric outcomes in breastfeeding populations. However, the present findings should be interpreted as exploratory observational outcomes and should not be interpreted as evidence of a direct physiological effect of maternal Pilates exercise on infant growth. Infant growth is a complex process influenced by a wide range of biological, nutritional, and environmental determinants. Consequently, the observed increases in infant weight and length should be interpreted with caution, and no direct causal relationship between the Pilates intervention and infant growth can be inferred.

According to the CVA, Mun and Roh^48^ examined how the Pilates program affected the FHP’s cervicothoracic sagittal alignment, muscle strength, and endurance. The findings indicated that the Pilates training enhanced the strength and muscular endurance in the shoulder, as well as cervicothoracic sagittal alignment.

Furthermore, ergonomic breastfeeding instruction decreased the frequency of musculoskeletal diseases in women, as shown by Afshariani et al.^49^. A study by Rani et al.^26^ evaluated common breastfeeding positions, recommended related to musculoskeletal problems and found that the most common position was the cross cradle hold associated with mechanical neck pain, while other positions also showed some musculoskeletal problems related to breastfeeding positioning, this is in line with the VAS results, which showed that the intervention group reported lower scores for pain.

Regarding FSS, a study by Saritacs and Picsirici^50^ investigated the effect of Pilates exercises on fatigue and physical self-perception in sedentary women, as for ten weeks, 50-minute Pilates sessions were held three days a week. The findings demonstrated that the Pilates exercise regimen can help sedentary women feel less exhausted and more confident about their bodies.

Although the current study showed notable improvements in FHP after the Pilates intervention, previous studies have reported inconsistent outcomes. For instance, Hürer et al.^16^ and Sharma and Roopa^40^ reported that Pilates programs were not superior to home exercises or cervical stabilization in improving pain and disability outcomes. These discrepancies could be explained by variations in the length of the intervention, the intensity of the exercise, the characteristics of the participants, the sample size, the degree of adherence, and the methods used to measure the results.

Numerous interrelated effects of the intervention are probably responsible for the observed improvements in CVA, cervical ROM, pain intensity, and QOL outcomes. Improved postural alignment may result from decreased overactivity of the cervical extensor and upper trapezius muscles and increased activation of the deep cervical flexor muscles. Improved neuromuscular control and joint mobility after repeated mobility-based and corrective exercises may account for increases in cervical ROM. Exercise-induced hypoalgesia, better muscle balance, and less mechanical stress on cervical tissues may all be linked to a decrease in pain intensity (VAS). Furthermore, decreased pain, higher confidence in carrying out daily tasks, improved functional capacity, and improved postural control are probably secondary to gains in QOL.

The observed improvements in posture, pain, and functional outcomes may be attributed to neuromuscular and biomechanical adaptations; however, these interpretations remain speculative as no direct electrophysiological or biomechanical measures were obtained in the present study. Therefore, the potential mechanisms discussed above should be interpreted with caution and viewed as plausible explanations rather than confirmed findings. Future studies incorporating objective neuromuscular and functional assessments are warranted to clarify underlying mechanisms.

Since FHP in females is thought to be linked to the onset and persistence of spine pain as well as several biomechanically driven disorders, studies have identified a substantial correlation between neck pain and this incorrect posture. The current study provides recent proof regarding the beneficial impact of Pilates activities on breastfeeding mothers’ FHP. Further research is necessary to assess the long-term impact of Pilates exercises on FHP in breastfeeding women because of its limitations, which include its brief length, the inability to assess FHP status before initiation of the breastfeeding experience, and the inability to examine the exercises’ long-term effects.

Several limitations should be taken into consideration, even if APECS posture assessment application provides a useful and accessible way to examine the CVA. Measurement variability may be introduced by participant position, camera distance, image quality, and lighting conditions, all of which affect app-based postural analysis. Although standardized imaging procedures were implemented to minimize these sources of variability, some degree of measurement error cannot be completely excluded. In addition, the APECS posture assessment application has been validated in general adult populations rather than specifically in postpartum or breastfeeding women. Postpartum-related changes, including alterations in body composition, breast enlargement associated with lactation, changes in thoracic posture, and reduced visibility of anatomical landmarks, may influence the accuracy of photogrammetric assessment. Therefore, the reliability reported in general adult populations may not be fully generalizable to breastfeeding women. Future studies should establish the validity, reliability, and other clinimetric properties of APECS specifically in postpartum and breastfeeding populations.

Another limitation is that examiner-specific intra-rater reliability for cervical ROM measurements was not formally established before data collection. Although all measurements were performed by the same trained assessor using a standardized protocol under identical testing conditions to minimize measurement variability, some degree of measurement error cannot be completely excluded. Future studies should establish and report examiner-specific intra- and inter-rater reliability to further strengthen measurement precision.

A limitation of the present study is the lack of detailed musculoskeletal assessments, including objective measures of muscle strength, muscle length, deep cervical flexor endurance, scapular muscle function, and core stability. Consequently, the specific physiological mechanisms contributing to the observed clinical and postural improvements could not be directly examined. Future research should incorporate comprehensive musculoskeletal evaluations to support more individualized exercise prescription and to provide a clearer understanding of the factors underlying treatment-related changes.

Another limitation of this study is that the control group received only breastfeeding positioning adjustment, which may not adequately control for therapist attention or placebo effects, potentially introducing performance bias. Therefore, the observed between-group differences should be interpreted with caution, as they may partly reflect non-specific effects of supervised intervention rather than the isolated effects of Pilates. Future studies should include attention-matched or sham-controlled interventions to better isolate the specific effects of the treatment. This study’s short follow-up duration and lack of long-term outcome evaluation are limitations that make it difficult to determine the long-term sustainability of the observed outcomes.

In addition, breastfeeding practices may naturally evolve between 6 and 12 months postpartum. Although daily breastfeeding duration was comparable between groups at baseline, changes in breastfeeding frequency and duration during the study period were not monitored and may have contributed to variability in postural loading. Future studies should consider stratification according to infant age and longitudinal monitoring of breastfeeding practices throughout follow-up.

An additional limitation of the present study is that sleep quality and depressive symptoms were not assessed using validated instruments. Both factors may influence pain perception, fatigue levels, breastfeeding self-efficacy, and QOL, and therefore their potential contribution to the observed outcomes cannot be disregarded. Future research should include standardized assessments of sleep and psychological well-being to enable a more comprehensive evaluation of their potential confounding effects.

Although the SF-36 is a multidimensional instrument with eight distinct subscales, the present analysis reported a composite score to summarize overall QOL. This approach may mask domain-specific effects and limit the clinical interpretability of the findings. Future studies should therefore present the SF-36 subscales separately to better capture intervention-related changes across physical and psychological domains.

A further limitation concerns the interpretation of infant growth outcomes. Although infants in the Pilates group demonstrated greater improvements in weight and length, infant growth is influenced by multiple factors that were not controlled in the present study, including genetic, nutritional, and environmental influences. Therefore, these findings should be considered exploratory and interpreted with caution, as the study was not designed to establish a causal relationship between maternal Pilates exercise and infant growth. Future studies should account for these potential confounding factors when evaluating infant growth outcomes.

Moreover, participant and therapist blinding was not feasible because of the nature of the exercise intervention. Although assessor blinding, allocation concealment, standardized treatment protocols, and independent statistical analysis were implemented to reduce potential bias, some degree of performance bias cannot be completely excluded.

Furthermore, there is an ethical limitation preventing the enrollment of a third group receiving Pilates exercise but not breastfeeding positioning adjustment.

## Conclusion

The results of this study suggest that incorporating Pilates exercises alongside breastfeeding positioning adjustment was associated with greater improvements in CVA, cervical mobility, pain, neck-related disability, fatigue, breastfeeding self-efficacy, QOL, and infant anthropometric measures among breastfeeding women with FHP compared with breastfeeding positioning adjustment alone. However, more research with longer follow-ups is necessary to confirm the reported effects and evaluate their long-term sustainability.

## Data Availability

The data generated during and/or analysed during the current study are available from the corresponding author on reasonable request.
